# Utilization of Oncotype DX in an Inner City Population: Race or Place?

**DOI:** 10.1155/2013/653805

**Published:** 2013-12-18

**Authors:** Amber A. Guth, Susan Fineberg, Kezhen Fei, Rebeca Franco, Nina A. Bickell

**Affiliations:** ^1^Division of Surgical Oncology, NYU-Langone Medical Center, 160 East 34th Street, 4th Floor, New York, NY 10016, USA; ^2^Department of Pathology, Montefiore Medical Center, Bronx, NY 10467, USA; ^3^Department of Health Evidence and Policy, Mount Sinai School of Medicine, New York, NY 10029, USA

## Abstract

Oncotype DX, a 21-gene-array analysis, can guide chemotherapy treatment decisions for women with ER+ tumors. Of 225 ER+ women participating in a patient assistance trial, 23% underwent Oncotype DX testing: 31% of whites, 21% of blacks, and 14% of Hispanics (*P* = 0.04) were tested. Only 3 white women were treated at municipal hospitals and none was tested. 3% of women treated in municipal hospital as compared to 30% treated at tertiary referral centers were tested (*P* = 0.001). Within tertiary referral centers, there was no racial difference in testing: 32% of whites, 29% of blacks, and 19% of Hispanics (*P* = 0.25). Multivariate analysis (model c-statistic = 0.76; *P* < 0.0001) revealed that women who underwent testing were more likely to have stage 1B (RR = 1.70; 95% CI: 1.45–1.85) and to be treated after 2007 (RR = 1.34; 95% CI: 1.01–1.65) and less likely to be treated at a municipal hospital (RR = 0.20; 95% CI: 0.04–0.94). Women treated at municipal hospitals were less likely to undergo testing resulting in a misleading racial disparity that is driven by site of care. As Oncotype DX can reduce overuse of chemotherapy, it is imperative to expand testing to those who could benefit from yet experience underuse of this test, namely, women treated at safety net hospitals. This trial is registered with NCT00233077.

## 1. Introduction

While women with early stage breast cancer frequently receive adjuvant chemotherapy to prevent recurrence, not all patients benefit from and require it.

Oncotype DX (ODX) (Genomic Health, Inc., Redwood City, CA) is a validated genomic predictor of outcome and response to chemotherapy in estrogen-receptor- (ER-) positive and node-negative breast cancer. ODX analyzes the expression of 21 genes within a tumor to determine a recurrence score that corresponds to a specific likelihood of breast cancer recurrence within 10 years of the initial diagnosis, as well as response to adjuvant treatment. Results are reported as a numeric recurrence score (RS) divided into low, intermediate, and high risk groups. Patients with a high RS derive additional benefit from chemotherapy to hormonal therapy, while those with a low score do not. Thus, the assay has the potential to enable women to avoid unnecessary chemotherapy.

The assay, costing approximately $4500, has been available since 2004, with Medicaid coverage becoming available in 2007. The American Society for Clinical Oncology and the National Comprehensive Cancer Network include the 21-gene signature assay in their guidelines for the management of lymph node-negative and ER-positive breast cancer [1, 2] The cost effectiveness of this assay in directing adjuvant systemic therapy has been established as well [3].

Most reports on ODX have examined the influence of recurrence score results on the use of adjuvant chemotherapy [3]. There is little data available on its use in minority and economically disadvantaged women. Ethnic differences in ODX have also not been well elucidated. This study describes our experience with the use of ODX in an inner city population.

## 2. Materials and Methods

374 women, with an early stage breast cancer surgically treated at 4 municipal hospitals and 4 tertiary referral centers in NYC between 2006 and 2009, participated in a randomized-controlled trial of patient assistance to reduce disparities in treatment [4, 5]. Their charts were abstracted to obtain pathology and treatment data. Of the 374 women, 225 had ER+ tumors stage 1B or greater, eligibile to undergo ODX testing. Bivariate comparisons were done with chi-square/*t*-tests and logistic regression models for multivariable comparisons. Race and municipal hospital were highly correlated, as only 3 white women went to municipal hospitals. Because there was no racial difference in testing within tertiary centers and only one woman in municipal hospitals got tested, we used hospital type rather than race in the final model.

## 3. Results

23% (52/225) of women underwent ODX. Women who got tested had earlier stage cancer and higher income and were white and treated at tertiary referral centers (see Table 1). There was no racial or insurance differences in receipt of ODX among women treated at tertiary referral centers. Of note, black and Hispanic women as compared to white women were more likely to be treated at municipal hospitals (29% versus 32% versus 3%; *P* < 0.0001). Clinical factors such as age and comorbidity and demographic factors such as insurance did not affect the likelihood of testing.

Multivariate analysis revealed that tested women were more likely to have stage 1B as compared to stage 2 (RR = 1.7, 95% CI: 1.45–1.85) and to be treated after 2007 as compared to 2006 or 2007 (RR = 1.34, 95% CI: 1.01–1.65) and less likely to get care at a tertiary referral hospital as compared to a municipal hospital (RR = 0.20; 95% CI: 1.01–0.94) (model c = 0.76; *P* < 0.0001).

Of women tested, 24/52 (46%) had a low recurrence risk, 19/52 (37%) had an intermediate score, and 9/52 (17%) had a high recurrence risk score, with no racial difference. All 9 patients at high risk (100%) were treated with chemotherapy; 58% at intermediate risk and only 4% at low risk were treated with chemotherapy.

## 4. Discussion

Despite advances in breast cancer care, disparities persist. Differences in tumor biology, health care access, and disease management all play a role [6]. For example, in a study of Medicare beneficiaries, Bach reported that black patients and white patients are to a large extent treated by different physicians, and the physicians treating black patients reported greater difficulties in obtaining access for their patients to high-quality subspecialists, high-quality diagnostic imaging, and nonemergency admission to the hospital [7].

We found that the two most important factors influencing ODX testing were tumor stage and receiving care at a municipal hospital. Importantly, site of care influenced testing. Women treated at municipal hospitals were unlikely to get ODX even after Medicaid would have covered the costs. In 2007, when Medicaid coverage became available for ODX, minority women at tertiary centers began to undergo testing; this was not true at municipal hospitals.

Our data suggest that the adoption of ODX was slower in municipal than in tertiary referral hospitals resulting in apparent racial disparity even after insurance coverage would have ensured equal access and could have saved unnecessary chemotherapy costs to patients and hospitals. Thus, the racial disparity in ODX testing reflected the site of care and not race since women were treated equally at the tertiary referral centers, with no racial disparity in those sites. These results reflect that diffusion of innovation lags at hospitals treating minority and underprivileged patients compared to tertiary referral centers.

In this study, chemotherapy decisions appear to be affected by ODX results. Responding to the biologic characteristics elucidated by the ODX, physicians were able to identify women with early stage tumors who could forego chemotherapy. Unfortunately only a small percentage of eligible women who could benefit were tested, and testing was limited to women treated at tertiary referral centers.

Our study is limited by the small sample size and its New York City locale which offers generous Medicaid benefits and may differ from states with more limited coverage.

## 5. Conclusion

As Oncotype DX influences treatment and can reduce overuse of chemotherapy, it is imperative to implement innovations that improve quality care at safety net hospitals. Such approaches have the potential to reduce racial and socioeconomic disparities in cancer care.

## Figures and Tables

**Table 1:** Patient characteristics of women ODX tested versus not tested.

	Total *N* = 225	Test done *N* = 52 (23%)	Test not done *N* = 173 (77%)	*P* value
Age (years), mean ± SD	55.9 ± 11.4	53.9 ± 9.9	56.5 ± 11.8	0.1615
Race				
White	110 (49%)	34 (65%)	76 (44%)	0.0438
Black	34 (15%)	7 (13%)	27 (16%)	
Hispanic	71 (32%)	10 (19%)	61 (35%)	
Low income (<$15,000/yr)	68 (33%)	10 (20%)	58 (37%)	0.0246
Insurance				
Commercial	126 (56%)	36 (69%)	90 (52%)	0.0902
Medicare	30 (13%)	5 (10%)	25 (14%)	
Medicaid/noninsured	69 (31%)	11 (21%)	58 (34%)	
Hospital type				
Tertiary referral center	188 (84%)	51 (98%)	137 (79%)	0.0013
Municipal hospital	37 (16%)	1 (2%)	36 (21%)	
Stage				
IB	112 (50%)	42 (81%)	70 (40%)	<0.0001
IIA	81 (36%)	8 (15%)	73 (42%)	
IIB	32 (14%)	2 (4%)	30 (17%)	
No positive nodes	144 (64%)	47 (90%)	97 (56%)	<0.0001
After 2007	150 (67%)	40 (77%)	110 (64%)	0.0736
